# Determination of the Real Cracking Moment of Two Reinforced Concrete Beams through the Use of Embedded Fiber Optic Sensors

**DOI:** 10.3390/s20030937

**Published:** 2020-02-10

**Authors:** Julián García Díaz, Nieves Navarro Cano, Edelmiro Rúa Álvarez

**Affiliations:** 1 School of Building Engineering, Universidad Politécnica de Madrid, 28040 Madrid, Spain; nieves.navarro@upm.es; 2 School of Civil Engineering, Universidad Politécnica de Madrid, 28040 Madrid, Spain; edelmiro.rua@upm.es

**Keywords:** Fiber Bragg grating, fiber optic sensors embedded in concrete, strain measurement, monitoring, cracking, weldable fiber optic sensors

## Abstract

This article investigates the possibility of applying weldable optic fiber sensors to the corrugated rebar in reinforced concrete structures to detect cracks and measure the deformation of the steel. Arrays have initially been designed comprised of two weldable optic fiber sensors, and one temperature sensor to compensate its effect in measuring deformations. A series of tests were performed on the structures to evaluate functioning of the sensors, and the results obtained from the deformation measures shown by the sensors have been stored using specific software. Two reinforced concrete beams simply resting on the support have been designed to perform the tests, and they have been monitored in the zones with maximum flexion moment. Different loading steps have been applied to the beams at the center of the span, using a loading cylinder, and the measurement of the load applied has been determined using a loading cell. The analysis of the deformation measurements of the corrugated rebar obtained by the optic fiber sensors has allowed us to determine the moment at which the concrete has cracked due to the effect of the loads applied and the deformation it has suffered by the effect of the different loading steps applied to the beams. This means that this method of measuring deformations in the corrugated rebar by weldable optic fiber sensors provides very precise results. Future lines of research will concentrate on determining an expression that indicates the real cracking moment of the concrete.

## 1. Introduction

It is fundamental to know the steel deformation in order to correctly calculate concrete structures. Although it deforms slightly before the concrete has not yet cracked, as shall be seen below, it acquires its greatest deformation when the concrete cracks, as it is not able to absorb traction. This article examines the appearance of the first cracks in reinforced concrete, embedding optic fiber sensors welded to the corrugated rebar steel, and it evaluates their deformation, comparing them with the values obtained from traditional material resistance calculation.

Although there is a lot of literature regarding crack detection in structural elements of reinforced concrete, the novelty of this article lies in the use of optic fiber sensors based on Bragg gratings (FBGs) welded to the corrugated steel rebars, which allows, on the one hand, determination of the precise moment when the crack appears, as the deformation of the steel shows a highly significant leap at the moment when it takes place, due to the concrete ceasing to collaborate in traction and the steel beginning to do so and, on the other hand, the deformation the steel suffers during the successive loading steps.

Diverse procedures have been used since methods have existed to detect cracks in reinforced concrete elements: infrared thermography [1,2,3], acoustic emission [4,5], fiber Bragg grating (FBG) [6,7,8,9,10,11,12], and digital image correlation [13,14]. One must emphasize the study of propagation and determination of the width of cracks in concrete, applying novel microwave sensors for crack detection in four reinforced concrete beam specimens [15]. By use of diffusing ultrasonic sensors, it has been possible to locate micro-cracks within the beam [16]. The stress wave technique with embedded smart aggregates in three samples of FRP reinforced concrete beams have provided satisfactory results in crack detection in the samples [17]. The use of plastic optical fibers to detect hairline cracks and ultimate failure crack in civil engineering structures has also obtained good results, even detecting the moment at which the structural element begins to crack and its evolution until the ultimate failure [18]. Carbon nanotube sensors embedded into concrete beams have also been used and were able to detect the initiation of cracks at an early stage of loads [19]. A novel sensing skin for monitoring cracks in concrete structures is capable of detecting, localizing and quantifying cracks in post-tensioned concrete specimens [20]. On the other hand, there are methods to determine cracks by images, such as the deep fully convolutional network (FCN). Images extracted from a video of a cyclic loading test on a concrete specimen is a reasonably method for crack detection [21], or use of a fully convolutional neural network [22]. A crack monitoring technique based on oblique fiber optic sensing network can accurately measure concrete cracks with a precision of 0.05 mm [23].

Fiber optic sensors based on Bragg gratings (FBGs) have been chosen for this study as they are more durable than conventional electric gages. They also provide long term signal stability and system stability, even under very unfavorable conditions, such as the vibration caused by roads. The cable length has no impact on the precision of the measurement. Multiplexing use allows different sensors to be placed on the same fiber optic cable, a much lighter cable than the conventional one of electric extensometric gages. The optic sensors are immune to electromagnetic and radiofrequency (EMI/RFI) interference, and resist hostile environments in the presence of water, salt, extreme temperatures, pressure (up to 400 bar), potentially explosive atmospheres and high voltage zones. Definitively, fiber optic sensors based on Bragg grating offer greater economic advantages, performance and precision than traditional electric gages [24].

FBG have been used both to detect cracks on the surface as well as by embedding them in the concrete. They have also been used to measure temperature and strain [25], biaxial-bending structural deformations [26], stress on the post-tensioned rod, detect moisture ingress in concrete based building structures [27].

In this study, we investigate the use of optic fiber sensors welded to the corrugated rebar to detect the moment at which the first crack takes place and the deformation the steel suffers from that moment onward during the whole process of loading the structural element. Section 2 analyses the materials used to perform the tests, the shape and dimensions of the beams studied. A tour is made through the characteristics of the equipment used and the software used. In Section 3, the test results are displayed and evaluated to determine the cracks and deformation of the steel in the two reinforced concrete beams, and conclusions are discussed in Section 4.

## 2. Materials and Methods

There are different devices on the market to measure deformations. The sensors chosen, within the group of Bragg grating (FBG) sensors, are weldable sensors, as these are to be integrated with the structural steel of the reinforced concrete structural elements to be tested.

The fiber optic sensors are highly temperature sensitive, so in order to compensate these effects, a temperature sensor will be included within the array. An array is a chain of sensors linked by a fiber optic cable; custom made for the structural element that is to be tested. The array to be installed on our beams shall contain two weldable deformation sensors and a temperature sensor. Each one of the deformation sensors shall be welded to the two bars that reinforce the beam under flexion and are to be stressed. As the temperature sensor only measures this, it will not be welded to the steel bars and will be located near to the deformation sensors.

Two beams of reinforced concrete are to be tested, with sections of 200 × 300 mm and 300 × 500 mm, and a length of 3000 mm. These sections have been chosen so they are sensitive to the loads to be applied.

### 2.1. Fiber Optic Sensors

**Figure 1:**
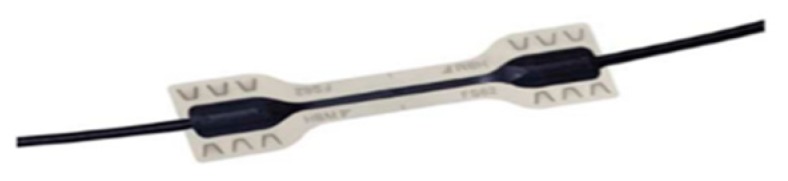
Weldable deformation sensor.

The fiber optic deformation sensors used are weldable (Figure 1). These are welded to the corrugated steel bars that constitute the reinforcement of the beam. Their position is that of the maximum effort that it will receive according to the different loading steps applied. Measurement of the deformation is reliably obtained in micrometers per meter.

**Figure 2:**
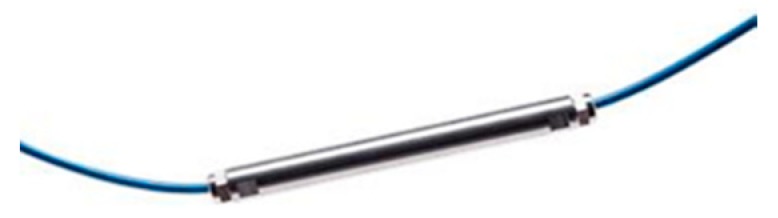
Temperature sensor.

The sensor chain was custom manufactured for each beam. Each chain has a temperature sensor (Figure 2) to compensate the temperature effect on the deformation sensors. The sensor chains have two terminals to which the interrogator may be connected. That redundancy effect is important as if a part of the chain were to be damaged, the terminal could always be measured in the undamaged area of the array.

### 2.2. Reinforced Concrete Beams

**Figure 3:**
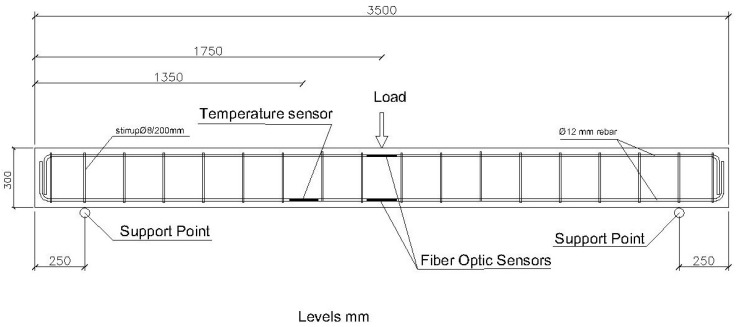
Reinforcement scheme of the 200 × 300 mm beam and application point of the loading steps.

**Figure 4:**
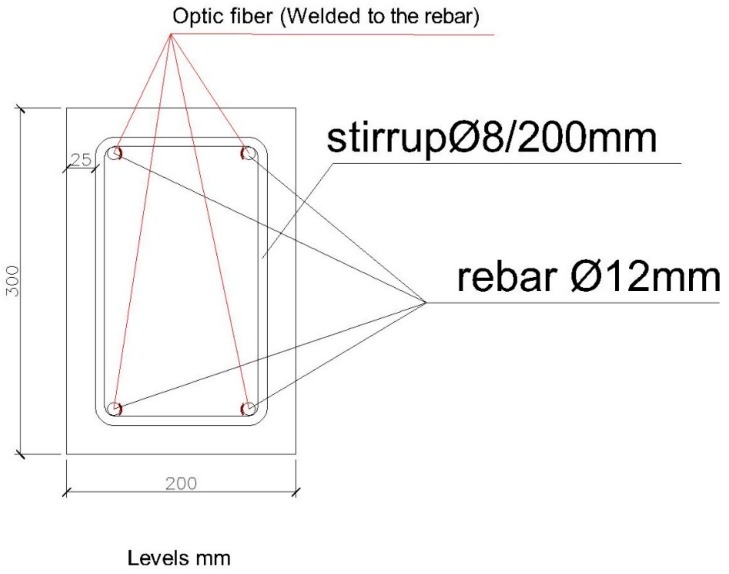
Beam section 200 × 300 mm.

The first structural element we are going to test is a reinforced concrete beam with a section of 200 × 300 mm. Its reinforcement is of four rebars with a diameter of 12 mm, with stirrups with a diameter of 8 mm every 200 mm. The steel quality is B500S. The beam length between resting points is 3000 mm. The reinforcement scheme and placement of the fiber optic sensors is illustrated in Figure 3 and Figure 4.

The choice of this beam with this section and reinforcement is due to the loads that are to be applied to it, so it is deformed significantly, and one may measure the deformations the steel suffers, as well as observe the cracks in the structural element, something that will be decisive in the section study.

The sensors are placed in the center of the beam, which is where the different loading steps are to be applied and where, as it is a beam that is simply resting on the support, there will be the maximum deformations in the structural element.

The temperature sensor has been placed attached to one of the corrugated steel bars, near the center zone, so it may compensate the effects the temperature has on the deformation sensors.

**Figure 5:**
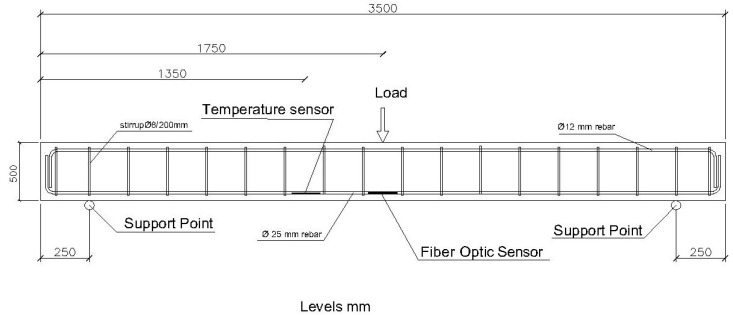
Reinforcement scheme of the 300 × 500 mm beam and application point of the loading steps.

**Figure 6:**
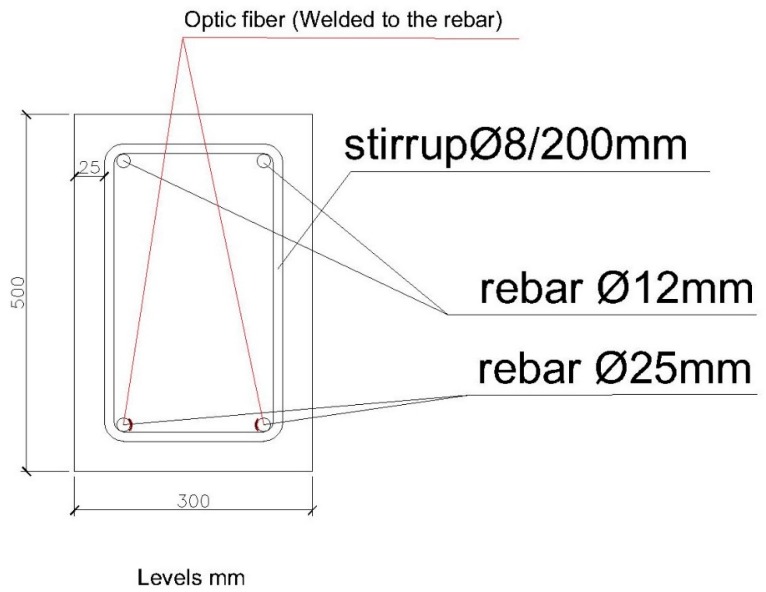
Beam section 300 × 500 mm.

The second structural element we are going to test is a reinforced concrete beam with a section of 300 × 500 mm. It is reinforced by two rebars with a diameter of 25 mm on the lower face and 2 rebars with a diameter of 12 mm on the upper face. The frames are rebar with a diameter of 8 mm, every 20 mm. As with the 200 × 300 mm beam, the length between support points is 3000 mm. Figure 5 and Figure 6 show schemes of the structural elements.

In this case, the sensors have only been placed on the lower bars, with a diameter of 25 mm, as the maximum deformation will take place on these and in the center of the span. As with the 200 × 300 mm beam, the temperature sensor has been placed near to the deformation sensors.

### 2.3. Design of the Experiment

In order to perform the experiment, the beams will be placed under different loading steps. The successive loads will be applied by a hydraulic press that will press on an RTN type loading cell of 10 Tn, with a ring torsion for the 200 × 300 mm beam. The device specifications are as follows: Nominal load: 10 t; Precision class: 0.05; Body measured: stainless steel; Protection class: IP68 to EN 60529; Cable type: shielded round cable, four wires in the case of the 200 × 300 mm beam, and for the 300 × 500 mm beam under RTN 100 Tn maximum ring torsion loading, Nominal load: 100 t; Precision class: 0.05; Body measured: stainless steel; Protection class: IP68 to EN 60529; Cable type: round shielded cable, four wires

Both loading cells are HBM brand (Hottinger Brüel & Kjaer Ibérica, S.L.U. San Sebastián de los Reyes, Madrid, Spain) with European Union Declaration of Conformity No. 238/2017-07 connected to an HBM QuantumX MX1615 data acquisition system with 16 channels, compatible with the following transducer technologies in all the channels:

- Extensometric gages on a circuit of 1⁄4, 1⁄2 with full bridge, variable bridge power (DC or bearing frequency of 1200 Hz), internal terminal resistance on 1/4 bridge (120 or 350 ohm); - Voltage (± 10 V);
- Pt100, resistance
- Potentiometer
- Sampling speed: max. 20 kS/s
- Automatic transducer identification: TEDS
- European Union Declaration of Conformity No. 263/2017-07.

In the process of successively loading the beam, the sag acquired by the structural elements will be measured by a linear potentiometer displacement transducer of 20 mm, 0.1% precision, compatible with MX1615B amplifier. The displacement transducer will also be connected to the QuantumX MX1615 data acquisition system.

The QuantumX MX1615 data acquisition system is connected to a computer in which software is installed to collect all the information, both from the loading cells as well as the displacement transducer. The software is Catman Easy, by the commercial brand HBM.

**Figure 7:**
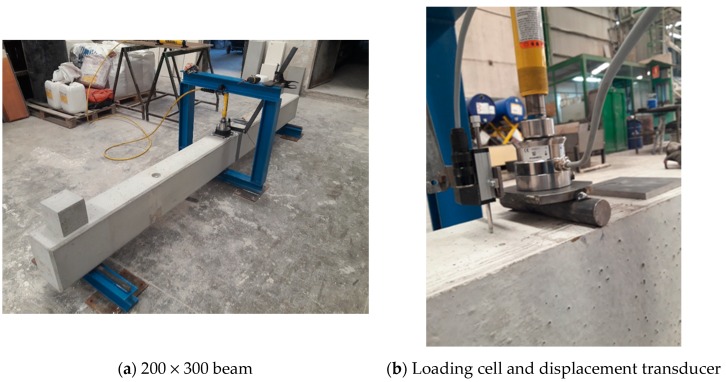
Loading cell and displacement transducer on the 200 × 300 mm beam.

Figure 7 shows the loading cell device and displacement transducer installed to perform the test, as well as the loading bridge with the beam located in the position to commence testing.

Data acquisition from the elements to measure applied force and deformation have been connected to the aforementioned QuantumX data acquisition system. Both the deformation as well as temperature sensors are connected to another device called interrogator. An optic interrogator is an optoelectrical instrument able to read fiber sensors with Bragg grating (FBG) in static and dynamic monitoring applications.

The same interrogator may obtain readings from an ample network of sensors of various types (deformation, temperature, displacement, acceleration, slope, etc.) connected through different fiber lines. All the data may be acquired simultaneously and with different sampling frequencies.

During the data acquisition, the interrogator measures the bandwidth associated with the light reflected by the optic sensors and converts it to technical units.

The interrogator model we are going to use is the FS22 (HBM), a device designed to interrogate Bragg grating based sensors. Its technology is continuous laser scan. This includes a reference to scannable bandwidth that provides continuous calibration and guarantees the long-term precision of the system. These interrogators are executed in an operating system in real time to acquire high quality data from a large number of sensors provided by the combination of broadband tuning range and simultaneous and parallel acquisition.

**Figure 8:**
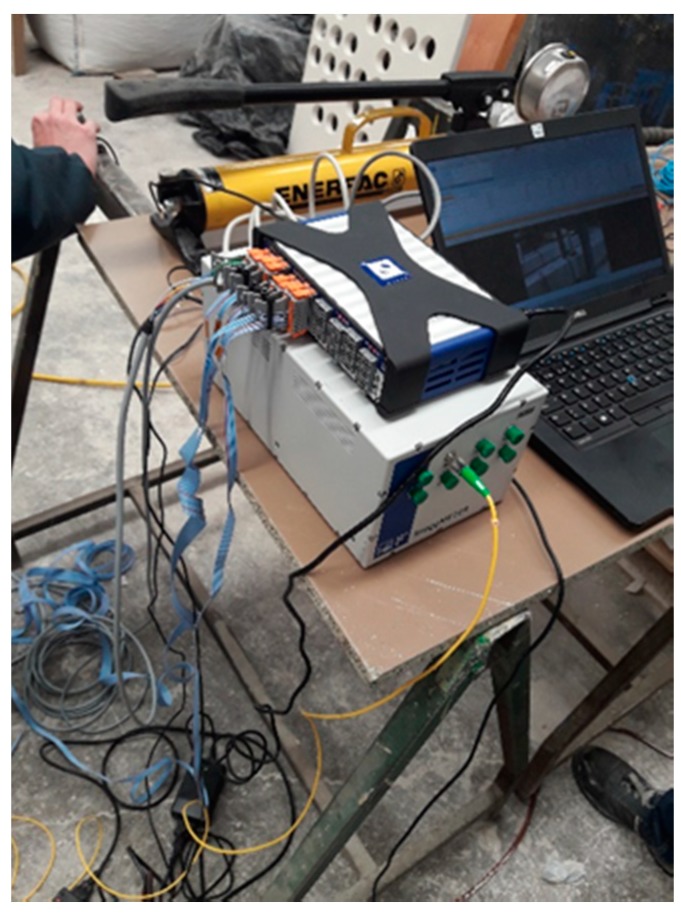
Data acquisition system installed. QuantumX, Interrogator and computer with Catman Easy software.

The interrogator is connected to the computer, which uses specific Catman Easy software, providing us the data on the deformations suffered by the beams in real time. The data acquisition system installed is shown in Figure 8.

## 3. Experimental Results and Discussion

The deformations we are going to measure are those of the steel, as that is the material the optic fiber is measuring. The cracking moment is a fundamental datum, as we know the moment at which the concrete ceases to absorb traction, in order for the steel to begin to work.

We must take into account, quoting Calavera [28] that “*Between the crack lips, the steel takes on the full traction strain on its own, but between the cracks, there is the anchorage of the reinforcement in the concrete and part of the traction force on the steel is transferred to it. If the traction on the concrete equals its resistance to traction, a new crack is formed*”.

**Figure 9:**
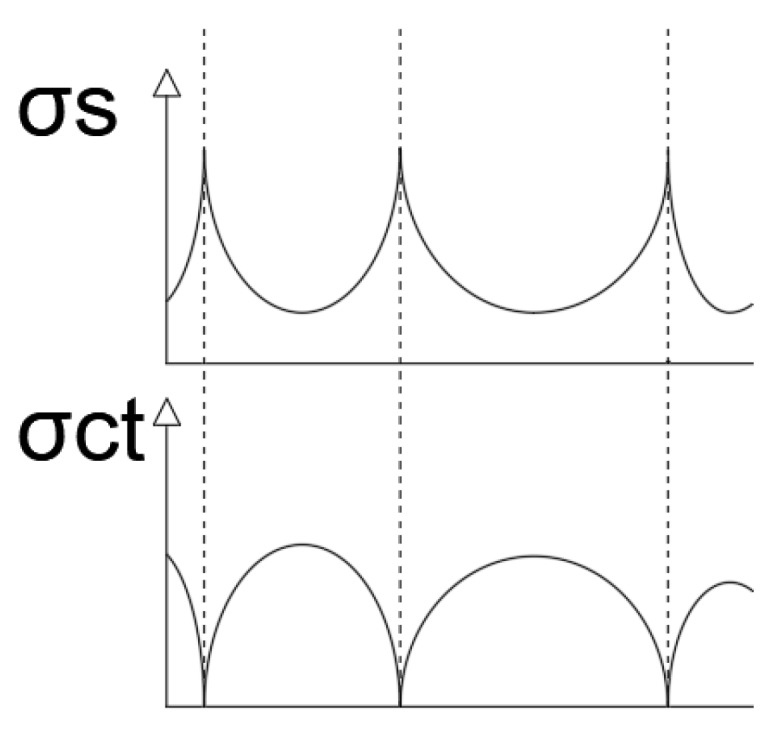
Variation in the tensions in concrete (σ_ct_) and steel (σ_s_) between cracks. In CALAVERA, J. (2008), Proyecto y cálculo de estructuras de hormigón. Tomo II, p. 372. (Designing and calculating concrete Structures, Volume II, p. 372). Give as [28].

This means that there is, between cracks, part of the concrete that absorbs deformations. At the exact point where there is a crack, the concrete does not collaborate and the whole deformation is absorbed by the steel. That fact is fundamental to understand how the structural element works (Figure 9).

**Figure 10:**
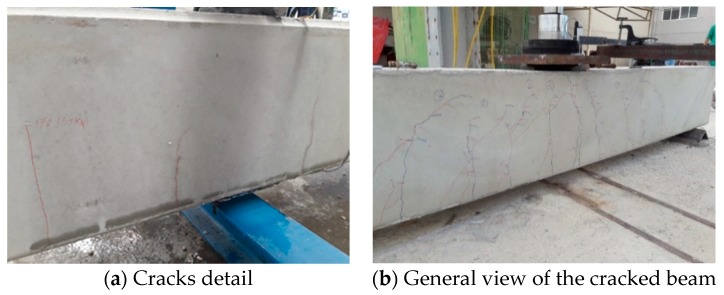
Visual inspection of cracks in 200 × 300 mm and 300 × 500 mm beams.

In the process of loading the structural elements, a visual inspection of the cracks that appear is carried out, to subsequently compare them with the theoretical calculations (Figure 10).

Laboratory test were carried out on the two beams studied, obtaining the following figures: For the 200 × 300 mm beam, the laboratory data is: Resistance to flexion-traction of concrete (f_ct_): 5.4 MPa; Resistance to compression of the concrete (f_ck_): 54.6 MPa; Concrete elasticity module (E): 33.400 MPa; and for the 300 × 500 mm beam: Resistance to flexion-traction of the concrete (f_ct_): 8.9 MPa; Resistance to compression of the concrete (f_ck_): 58.8 MPa; Elasticity module of the concrete (E): 33.053 MPa.

We used the values obtained in the laboratory for resistance of flexion-traction of the concrete (f_ct_) to calculate the cracking moment. The cracking moments are calculated by applying the Equation (1), obtaining the following results:

M_fis_ = f_ct_ * (b * h^2^)/6 (1)

For the 200 × 300 mm beam this gave M_fis_ = 16.2 mkN, and for the 300 × 500 mm beam: M_fis_ = 111.25 mkN. Once these values were known, the beams underwent different loading steps, applied in the center of the span, linking the loads applied to the deformations caused in the corrugated steel according to the tables included in the relevant sections. Visual inspections were performed to control the moment when the first cracks appear in these.

**Figure 11:**
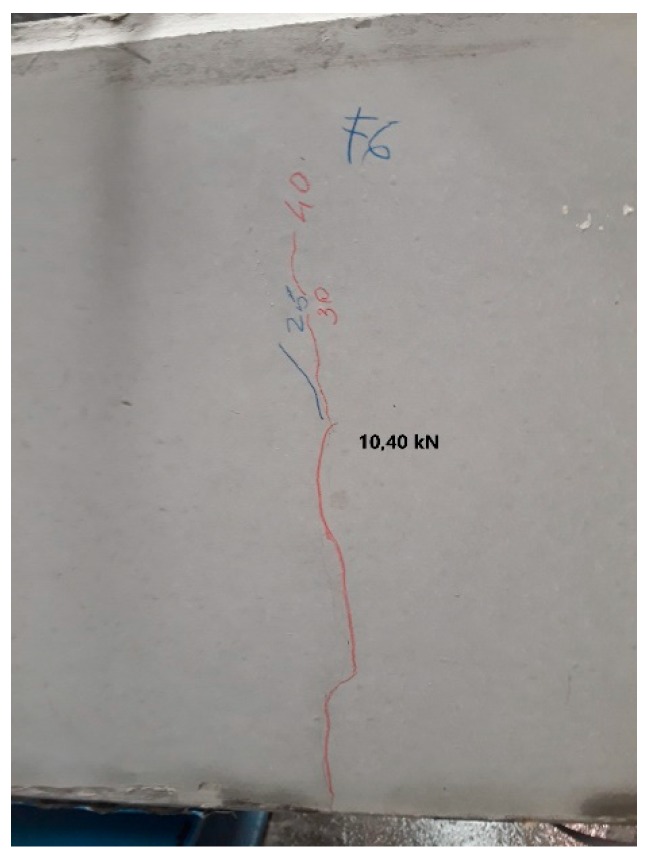
Visual inspection of cracks on the 200 × 300 mm beam.

If we analyze the cracks in the section, and when they take place, we observe that these have taken place much before the cracking moment obtained by calculation. For the 200 × 300 mm beam, it is observed that the cracks nearest to the center of the span take place with a load of 10.4 kN, and a moment of 7.80 mkN, which is much further from the theoretical value obtained. The value of 10.4 kN has been obtained by visual inspection (Figure 11), but as we shall see, the real load for the beam to begin to crack is 8.5 kN.

**Figure 12:**
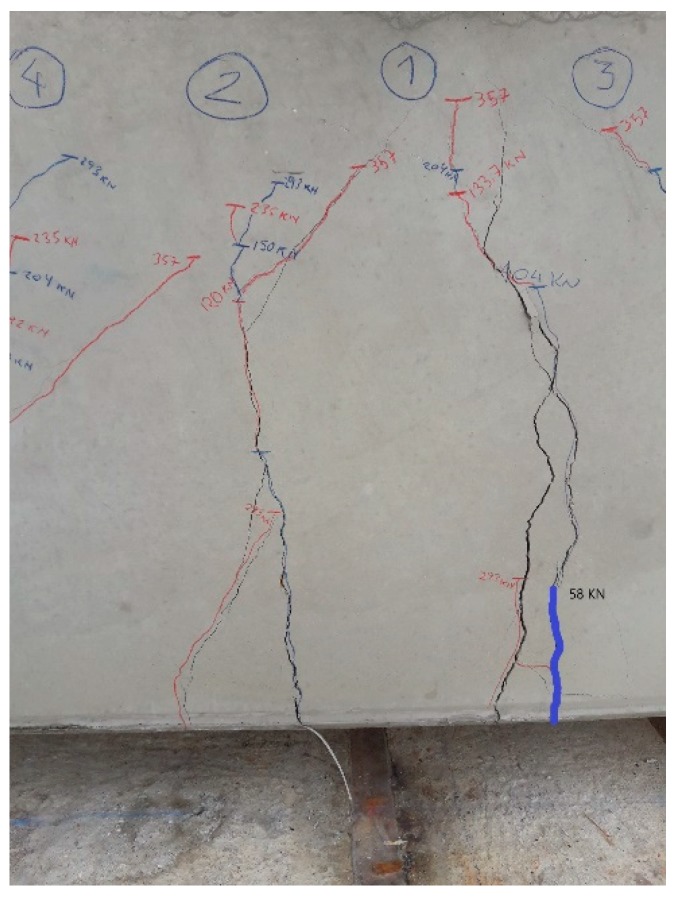
Visual inspection of cracks on the 300 × 500 mm beam.

In the case of the beam of 300 × 500 mm, although the cracking moment obtained by calculation is 111.25 mkN, the real cracking moment is 40.5 mkN, that corresponds to a load of 58 kN, as we may see below (Figure 12).

### 3.1. 200 × 300 mm Beam

**Figure 13:**
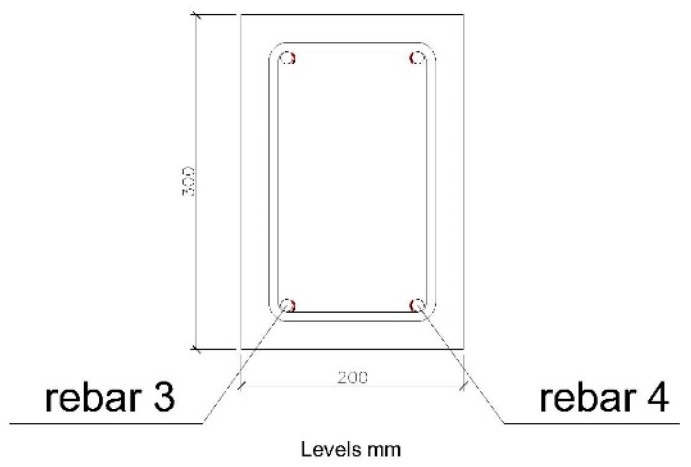
Position of the rebars studied in the 200 × 300 mm beam.

The section studied is shown in Figure 13. The corrugated steel bars correspond to those located in the lower part of the beam, which will be subject to traction.

#### 3.1.1. Determining the Moment When the Crack Takes Place

The loads began to be applied in the center of the beam span, within an interval ranging from 0.56 kN to 42.24 kN. Using Catman software, we obtained the deformation that takes place in corrugated steel bars according to the loads applied in Table 1.

**Figure 14:**
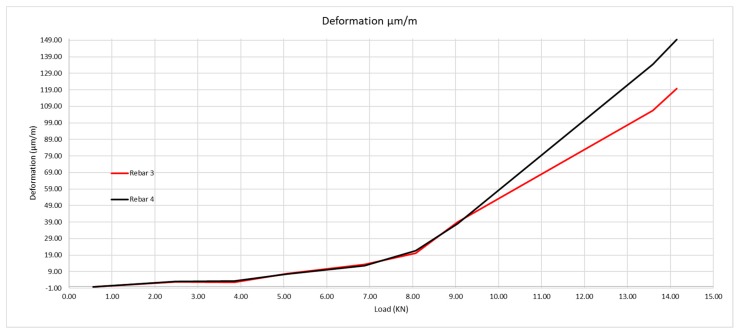
Deformation of rebars according to the loads applied.

As shown in Figure 14, the steel deformation is about five or six µm/m for increases in load between one and two kN, while it is 20 µm/m when the load is from 8.07 to 9.05 kN.

These results imply the need to reconsider the moment of the concrete cracking and the steel deformation, as the moment of real cracking is less than that obtained by calculation as well as by visual inspection. It is evident that micro-cracks that are invisible to the human eye are formed, but that the optic fiber is able to detect. One may thus determine that a load of 8.5 kN is what makes the concrete crack.

#### 3.1.2. Steel Deformation with Laboratory Data on the Concrete Compared with Steel Deformation Obtained by Optic Fiber Sensors

A comparison shall be made between the theoretical deformation obtained by calculation and the real deformation process indicated by the sensors. To that end, a concrete with the characteristics obtained in the laboratory has been created, with a resistance to traction of 5.4 MPa, which provides a cracking moment of 16.2 mkN. Although all the moments acting have been checked in FAGUS, Table 2 shows the deformation of the steel for the moment of 31.68 mkN.

**Figure 15:**
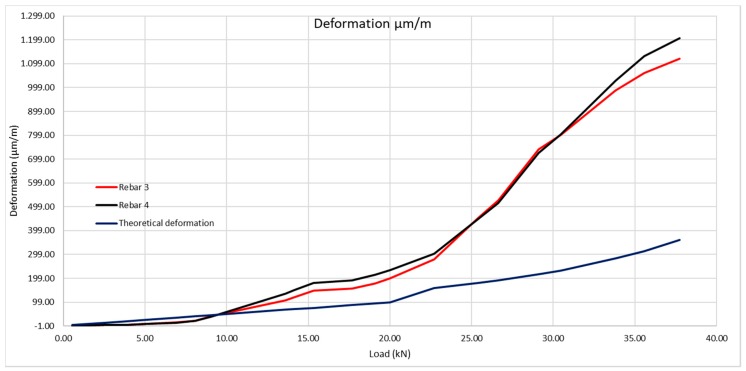
Theoretical deformation of the rebars compared with real deformation obtained with the optic fiber sensors.

The rest of the deformation values are shown in Table 3 and Figure 15.

It is evident that the deformation of the steel is in fact greater than what is stated in the theoretical calculations. This is caused by cracking of the beam that, as aforementioned, happened before it was expected.

#### 3.1.3. Deformation of the Steel with the Real Cracking Moment of the Concrete Compared with the Deformation Obtained Using Optic Fiber Sensors

We shall now see how the steel in the beam is deformed at theoretical level with the real data for traction resistance of the concrete and the real cracking moment. We have already noted that the beam cracks under a load of 8.5 kN, which corresponds to a cracking moment of 6.38 mkN. With that moment, and applying Equation (1) given above.

We obtain the real resistance to traction of the concrete, that shall be 2.13 MPa. With that data, we input the value in the characteristics of our concrete in the FAGUS program. The program provides a value of the steel deformation in the crack, and another in the uncracked concrete. The section studied is between two cracks, so that figure must be averaged. Table 4 provides the values with and without cracking, for a moment of 28.31 mkN. The rest of the values have been obtained the same way.

**Figure 16:**
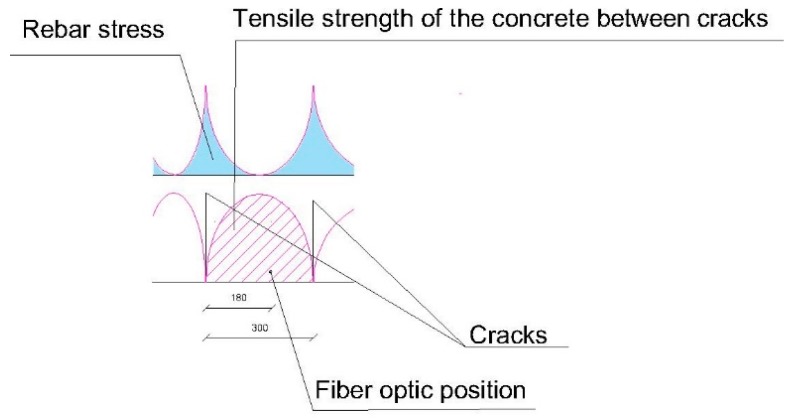
Determining deformation of the steel between cracks on 200 × 300 mm beam.

In Figure 16 one observes, on the one hand, the position of the optic fiber, the distance between cracks, which is 300 mm, and the position related to the crack on the left side of the optic fiber, which is 180 mm. That means that the concrete between fissures contributes to traction of the beam, and to the steel becoming deformed, but not if it is in the crack.

Table 5 includes the steel deformation without cracking and the steel deformation with cracking. The deformation adopted shall be an interpolation between both figures.

**Figure 17:**
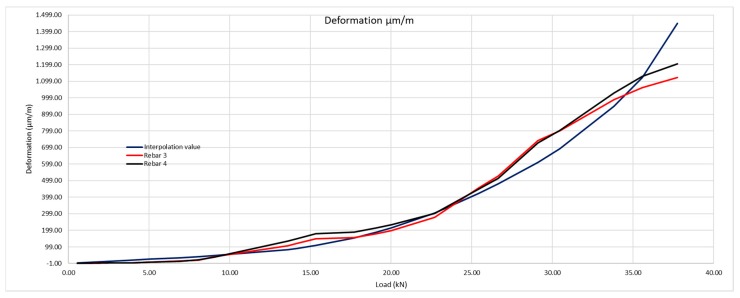
Theoretical deformation of the rebars with its real resistance to traction of 2.13 MPa compared with the real deformation obtained with the optic fiber sensors.

Considering these results, Figure 17 shows that the theoretical deformation of the steel is in keeping with that obtained by the optic fiber sensors. The contribution by the concrete between cracks plays an important role in determining the steel deformation.

We observe that the theoretical deformation value interpolated grows over a soft curve. This is due to the sensor being approximately in the center between cracks, which makes the concrete between cracks contribute to less deformation of the steel than if the sensor were to be very near to a crack or in the actual crack. That is precisely what happens in the following beam studied, where we observe that the interpolation curve suffers a major leap at the moment of the cracks taking place.

**Figure 18:**
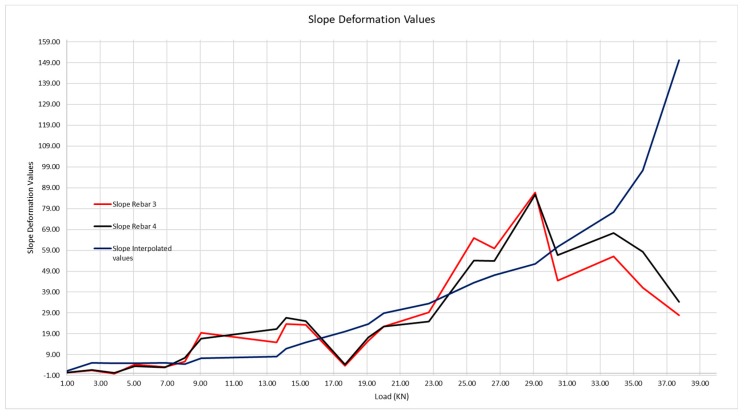
Slope curves of the theoretical deformation of the corrugated steel (interpolated values) and of rebars 3 and 4.

In order to be able to determine the precise moment when the crack takes place and how this grows by application of the successive loads, we shall transform Figure 17 into a graphic, Figure 18 that shows, in an equivalent manner to Figure 17, the deformation slope curves according to the loads. The greater the slope, the greater the deformation.

In Figure 18 we observe how the first significant leap in loading takes place, which is in the interval [8.07;9.05], that is, that the first crack begins to form at a load of 8.07 kN, and it cracks until reaching 9.05 kN. The slope values of these curves are shown in Table 6.

**Figure 19:**
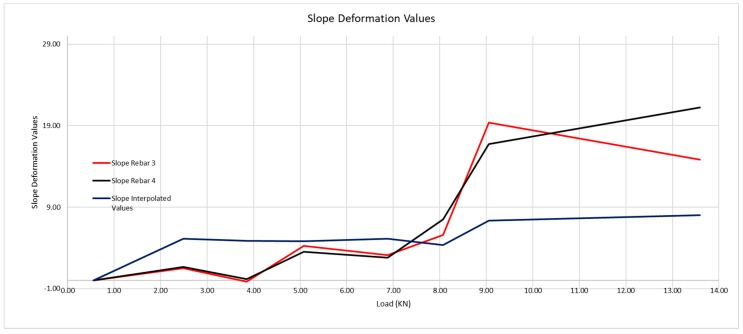
Detail of the first crack forming in the 200 × 300 mm beam.

It is evident that concrete is a material regarding which we cannot do more than approach its behavior by experience, although with embedded sensors we are able to know the moment at which the material cracks, and with that result, know its behavior much better. Figure 19 shows the interval in which the crack arises in greater detail.

One may see that, while the beam has not cracked, the steel is gradually deformed, on the contrary to what traditional structure calculation theory says as, in this, the traction is absorbed by the concrete, a fact that is proven not to be the case in these graphs. Once the cracks start, when we go from 8.07 to 9.05 kN applied load, the deformation of the steel is much more significant, as the concrete contributes to a lesser extent to absorb the traction. The greater or lesser contribution by the concrete depends on the position of the sensor between the cracks.

### 3.2. 300 × 500 mm Beam

**Figure 20:**
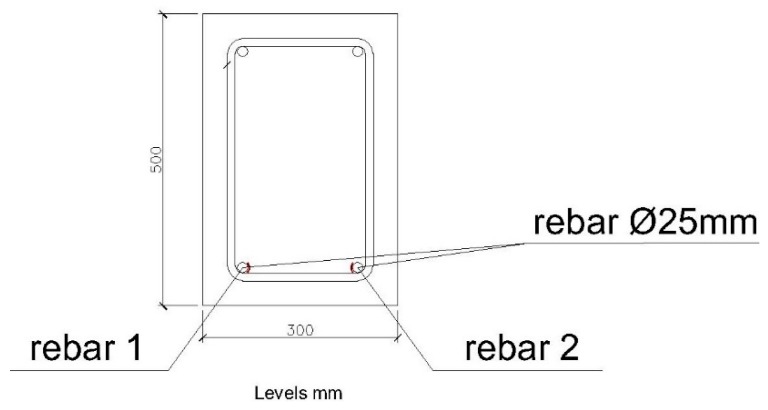
Position of bars studied in 300 × 500 mm beam.

The section of the beam that will be analyzed below is shown in Figure 20. The corrugated steel bars studied correspond to the lower part of the beam and are those that will be placed under traction.

#### 3.2.1. Determination of the Moment the Crack Takes Place

Loading begins in the center of the beam span, at an interval ranging from 5.20 kN to 275 kN. After applying these loads, and using Catman software, we obtain the deformation that takes place in the corrugated steel bars according to the loads applied (Table 7).

**Figure 21:**
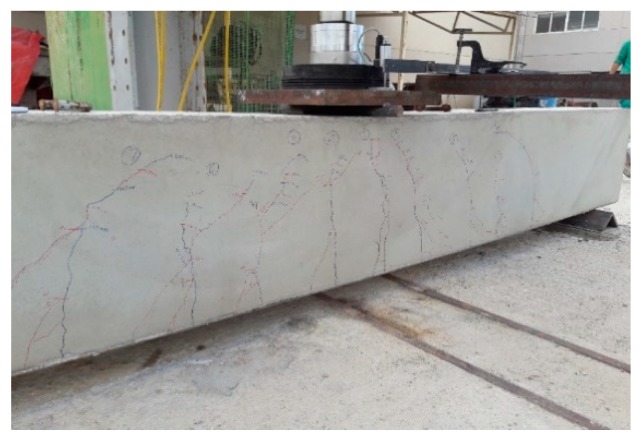
Visual control of cracking in the 300 × 500 mm beam.

Once more, the beam has cracked before reaching the calculated cracking moment. The cracking process of the beam was observed, as with the 200 × 300 mm beam, obtaining perceptible cracking on visual inspection with a load of 58 kN. Figure 21 shows the visual control of cracking of the beam throughout the whole process.

**Figure 22:**
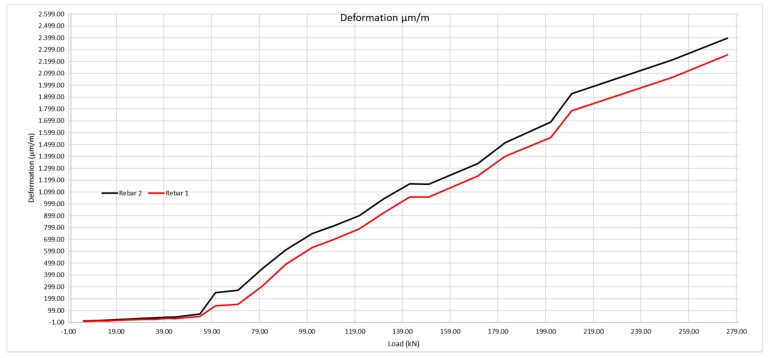
Deformation of the rebars 1 and 2 according to the loads applied.

In Figure 22, we may observe the moment at which the beam cracks, as the steel deformation grows in an obvious manner. The loading at which the cracking takes place, according to the deformation measure obtained by the optic fiber is 54 kN, compared with the 58 observed in the visual inspection. Thus, it would be those 54 kN that would be taken as the loading value to calculate the cracking moment.

#### 3.2.2. Steel Deformation Using the Concrete Laboratory Data Compared with Steel Deformation Obtained From the Optic Fiber Sensors

The steel deformation shall first be analyzed using the data obtained in the laboratory, compared with that provided by the optic fiber. To do so, a concrete has been created with the characteristics described above, with fct = 8.9 MPa, and M_fis_ = 111.25 mkN.

The steel deformation is obtained using the program FAGUS. We shall show the result provided by FAGUS for a moment of 127.88 mkN, the rest of the results having been obtained in the same way (Table 8).

**Figure 23:**
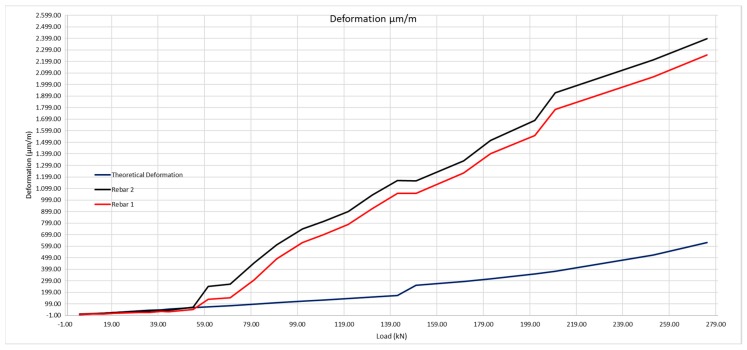
Theoretical deformation of the rebars compared with the real deformation obtained using the optic fiber sensors.

The same method shall be applied as for the 200 × 300 mm beam, considering the steel deformation for resistance to traction of the concrete obtained by the laboratory tests, and subsequently that obtained with the optic fiber (Table 9 and Figure 23).

#### 3.2.3. Deformation of the Steel at the Real Cracking Moment of the Concrete Compared with Deformation of the Steel Obtained Using Optic Fiber Sensors

**Figure 24:**
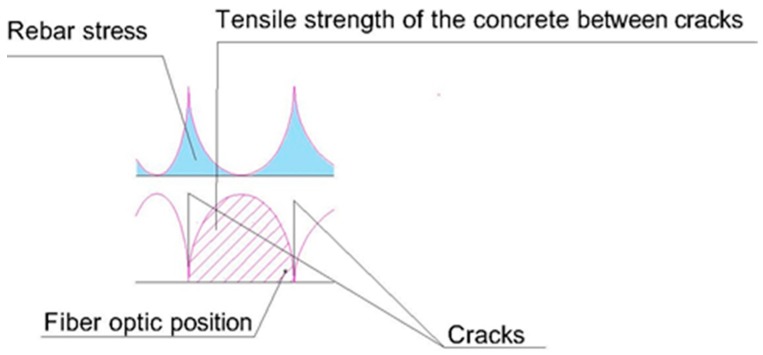
Determining deformation of the steel between cracks on 300 × 500 mm beam.

For a load of 54 kN, the moment corresponds to 40.5 mkN, and applying Equation (1) we obtain a resistance to traction of the concrete of 3.24 MPa, compared with the 8.9 MPa obtained in the laboratory. In this case, the crack has opened at a point very near to the optic fiber, so the concrete can barely contribute to avoid deformation of the steel (Figure 24).

We see what happens when the resistance to traction of the concrete with which we perform the calculations using the FAGUS program corresponds to the 3.24 MPa. Table 10 shows the deformation of the corrugated steel for a moment of 127.88 mkN.

Table 11 includes the deformation of the steel without cracking, and the deformation of the steel with cracking. The deformation adopted shall be an interpolation of both values.

**Figure 25:**
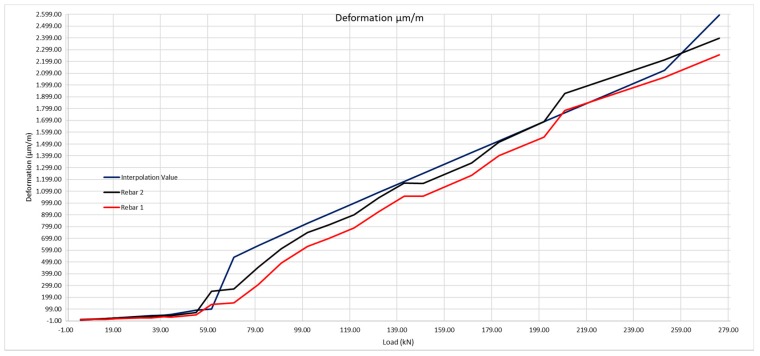
Deformation of the rebars with its Resistance to real traction of 2.13 MPa compared with real deformation obtained using optic fiber sensors.

Figure 25 is the graphic representation of figures in Table 11.

We observe that by inputting the behavior real values of the concrete, the beam deformation curve corresponds to the data provided by the optic fiber sensors. In this case, as the optic fiber is very near to a crack, there is a sharp leap at the moment it takes place. As we shall see in the slope figures, these are much steeper than in the previous case, in which the optic fiber was approximately in the center between the cracks, which caused these slopes to be less steep. The slope values of these curves are recorded in Table 12.

**Figure 26:**
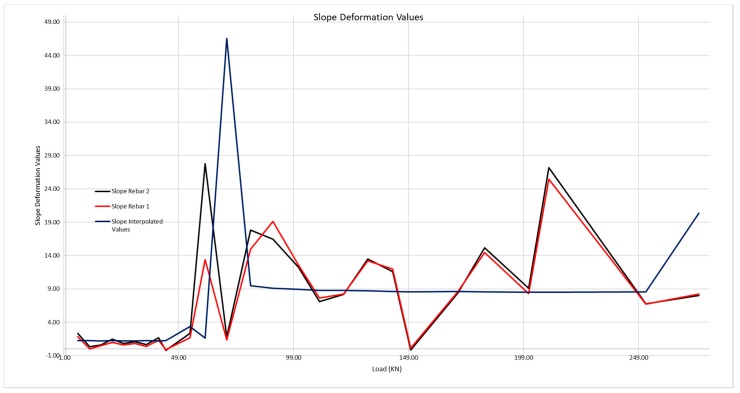
Slope theoretical deformation curves of the rebars (interpolated values) and of rebars 1 and 2.

In Figure 26, we observe how there is a significant first leap at a load that is in the interval [54.00;60.54], that is, that the first crack begins to form with a load of 54 kN, and it cracks until reaching 60.54 kN.

**Figure 27:**
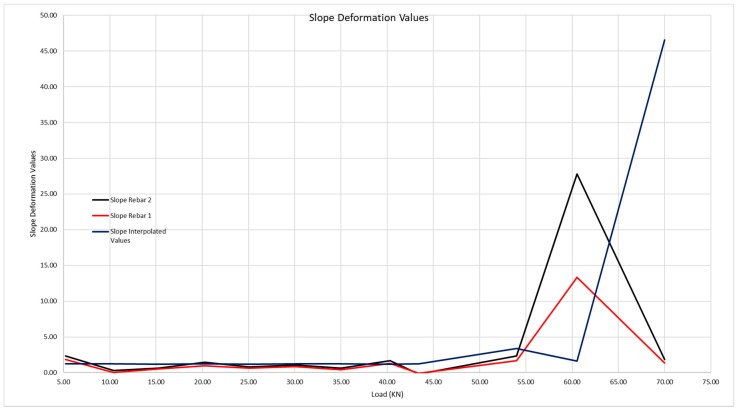
Detail of the first crack forming in the 300 × 500 mm beam.

Figure 27 shows the interval within which the crack takes place in greater detail

The moment at which the crack in the beam takes place is evident, as the deformation of the steel grows evidently. The load at which the crack takes place is 54 kN, compared with the 58 kN observed during the visual inspection.

As happened with the 200 × 300 mm beam, the theoretical deformation of the beam reinforcement is far from appearing like its real behavior. In this case, the crack has opened up at a point very near to the optic fiber, so the concrete shall not collaborate when its traction tension is reached.

In this case, we must observe what happens at the loading point of 54 kN, where the slope of the curve is much steeper, something that did not happen in the graph of beam 200 × 300 mm. This is due to the crack having opened very near to the location of the optic fiber, so that, in analysis of the section, the concrete barely collaborates in traction of the beam, and practically all the traction is borne by the steel.

It is noted that the optic fiber shows us the precise moment at which the beam cracks, that the cracks open much earlier than what the laboratory tests say, and that taking the data provided by the optic fiber, we may determine the behavior of the structural element in a much more precise way.

It is evident that when calculating a structural element, we do not know what will happen to it, when the piece will really crack. Considering an expression that draws that value closer to reality shall be a matter to be studied in future lines of investigation.

## 4. Conclusions

Two beams with a rectangular section in which fiber optic sensors were embedded have been tested to analyze the real deformation of the steel when they are submitted to different loading stages. 

Appearance of the first cracks has been observed in both cases. These appear much earlier than the calculation predicts. The appearance of the first cracks is a fundamental matter to understand the real behavior of the structures. Fiber optic sensors were used to observe how a sudden change in deformation of the steel takes place. Moreover, with the advantage of the measurements being in real time, a fact that provides greater value to evaluation of the structural health of the elements tested. It is evident that this sudden change leads to it being deformed to greater extent as a consequence of the concrete cracking.

Thus, considering the results obtained, we may know the precise moment at which the beam cracks through embedded fiber optic sensors. On studying the deformations, it has been noted that even when test pieces extracted at the moment of concrete pouring were tested, and they were tested on the day when the tests were to be carried out, these values do not match the behavior of the concrete under traction. In both cases, the beam cracked much before the laboratory tests indicated.

Thus, placement of sensors welded on corrugated steel bars within reinforced concrete structural elements, at their maximum effort points, is a precise, reliable method to determine the moment at which the first cracks take place, as it has been possible to prove according to the results obtained.

After ascertaining the real cracking moment of the concrete, we precisely obtained its resistance to traction and, thus, the real deformation of the corrugated steel during application of the loads.

As stated in the introduction, the existing studies on concrete cracking use diverse methods to detect cracks. As is known, concrete is a material that resists compression well, but that is not the case with traction efforts. The reinforcement of the structural elements is placed, among other reasons, to bear the traction the concrete is not able to bear. The method proposed herein provides, as a novelty, detection of cracks that is observed thanks to the optic fiber sensors welded to the corrugated steel bars, at the precise moment when the steel begins to deform significantly. This causes a leap in its deformation, which is detected for the relevant load applied. Moreover, once the steel begins to deform, it is possible to know the deformation it will suffer during the whole period of application of the different loading steps to which the structural element is submitted. It has been proven that the deformation of the steel measured with optic fiber sensors corresponds to the theoretical values of the traditional materials resistance calculation, as long as the real cracking moment of the concrete is taken as the starting point.

We may conclude, considering these graphs obtained from the experiment carried out, on the one hand that laboratory tests to determine flexion-traction resistance of concrete provide very conservative results, that have nothing to do with what really happens in the structural element. And on the other, that deformation of the steel, obtained with these tests, are quite far from its real behavior. This method of evaluating the structural health of a simple element of reinforced concrete may be transferred to more complex structural elements of buildings in construction to know the behavior of the structure when the formwork removal takes place and their actual weight begins to bear down on the structures, and subsequently the application of deadweight and overburdens in use, when the building is put into operation.

A monitored building may provide us information on what overburdens it is able to bear, a highly important factor when one wishes to change the use of a building and the overburdens it is to be subject to are higher than those initially designed. In this case, and according to the data obtained, one might even be able to avoid possible structural reinforcement, as we would know what the building may really bear, with the financial savings that would involve.

## Figures and Tables

**Table 1:** Relation between loads applied and deformation of the rebars 3 and 4.

Load (kN)	Moment (mkN)	Rebar Deformation 3 (µm/m)	Rebar Deformation 4 (µm/m)
0.56	0.42	−0.18	−0.17
2.49	1.87	2.71	3.02
3.85	2.89	2.54	3.28
5.08	3.81	7.79	7.61
6.88	5.16	13.4	12.70
8.07	6.05	20.05	21.61
9.05	6.79	39.05	38.03
13.59	10.19	106.46	134.48
14.15	10.61	119.68	149.43
15.34	11.51	147.52	179.24
17.69	13.27	156.19	189.27
19.09	14.32	177.99	213.38
20.01	15.01	198.74	234.04
22.71	17.03	277.62	301.36
25.42	19.06	453.07	447.94
26.64	19.98	526.35	513.97
29.11	21.83	740.68	725.88
30.45	22.84	800.39	802.02
33.80	25.35	988.09	1027.15
35.57	26.67	1060.50	1130.08
37.75	28.31	1121.17	1205.10

**Table 2:** Deformation of the rebars 3 and 4 for a moment of 31.68 mkN by FAGUS.

Formula/Result	Name	Max.	Min	Value
ε Rebar 3	Rebar 3	0.3414	0.3414	‰
ε Rebar 3 Value in crack	Rebar 3	0.4878	0.4878	‰
ε Rebar 4	Rebar 4	0.3414	0.3414	‰
ε Rebar 4 Value in crack	Rebar 4	0.4878	0.4878	‰

**Table 3:** Theoretical deformation of the rebars 3 and 4 for M_fis_ = 16.2 mkN and f_ct_ = 5.4 MPa with FAGUS.

Load (kN)	Moment (mkN)	Theoretical Deformation (µm/m)	Deformation Rebar 3 (µm/m)	Deformation Rebar 4 (µm/m)
0.56	0.42	2.60	−0.18	−0.17
2.49	1.87	12.50	2.71	3.02
3.85	2.89	19.10	2.54	3.28
5.08	3.81	25.00	7.79	7.61
6.88	5.16	34.20	13.4	12.70
8.07	6.05	39.40	20.05	21.61
9.05	6.79	44.70	39.05	38.03
13.59	10.19	66.90	106.46	134.48
14.15	10.61	69.50	119.68	149.43
15.34	11.51	75.40	147.52	179.24
17.69	13.27	87.20	156.19	189.27
19.09	14.32	93.70	177.99	213.38
20.01	15.01	98.30	198.74	234.04
22.71	17.03	158.80	277.62	301.36
25.42	19.06	179.80	453.07	447.94
26.64	19.98	190.40	526.35	513.97
29.11	21.83	215.10	740.68	725.88
30.45	22.84	231.10	800.39	802.02
33.80	25.35	282.10	988.09	1027.15
35.57	26.67	313.60	1060.50	1130.08
37.75	28.31	359.10	1121.17	1205.10

**Table 4:** Deformation of rebars 3 and 4 for a moment of 28.31 mkN by FAGUS.

Formula/Result	Name	Max.	Min	Value
ε Rebar 3	Rebar 3	1.3895	1.3895	‰
ε Rebar 3 Value in crack	Rebar 3	1.9850	1.9850	‰
ε Rebar 4	Rebar 4	1.3895	1.3895	‰
ε Rebar 4 Value in crack	Rebar 4	1.9850	1.9850	‰

**Table 5:** Deformation of the rebars 3 and 4 for M_fis_ = 8.5 mkN and f_ct_ = 2.13 MPa with FAGUS.

Load (kN)	Moment (mkN)	Deformation without Crack (µm/m)	Cracking Deformation (µm/m)	Interpolation Value (µm/m)	Deformation Rebar 3 (µm/m)	Deformation Rebar 4 (µm/m)
0.56	0.42	2.60		2.60	−0.18	−0.17
2.49	1.87	12.50		12.50	2.71	3.02
3.85	2.89	19.10		19.10	2.54	3.28
5.08	3.81	25.00		25.00	7.79	7.61
6.88	5.16	34.20		34.20	13.4	12.70
8.07	6.05	39.40		39.40	20.05	21.61
9.05	6.79	44.70	63.70	46.60	39.05	38.03
13.59	10.19	79.50	113.60	82.91	106.46	134.48
14.15	10.61	85.90	122.80	89.59	119.68	149.43
15.34	11.51	102.80	146.90	107.21	147.52	179.24
17.69	13.27	147.80	211.10	154.13	156.19	189.27
19.09	14.32	179.70	256.80	187.41	177.99	213.38
20.01	15.01	205.20	293.10	213.99	198.74	234.04
22.71	17.03	291.70	416.70	304.20	277.62	301.36
25.42	19.06	404.30	577.50	421.62	453.07	447.94
26.64	19.98	459.50	656.40	479.19	526.35	513.97
29.11	21.83	583.80	834.00	608.82	740.68	725.88
30.45	22.84	662.10	945.80	690.47	800.39	802.02
33.80	25.35	910.10	1300.10	949.10	988.09	1027.15
35.57	26.67	1075.10	1535.80	1121.17	1060.50	1130.08
37.75	28.31	1389.50	1985.00	1449.05	1121.17	1205.10

**Table 6:** Slope values of the theoretical deformation curves and of rebars 3 and 4.

Load (kN)	Interpolation Value (µm/m)	Deformation Rebar 3 (µm/m)	Deformation Rebar 4 (µm/m)	Slope Interpolation Value	Slope Rebar 3	Slope Rebar 4
0.56	2.60	−0.18	−0.17	0.00	0.00	0.00
2.49	12.50	2.71	3.02	5.13	1.50	1.66
3.85	19.10	2.54	3.28	4.85	-0.13	0.19
5.08	25.00	7.79	7.61	4.80	4.27	3.52
6.88	34.20	13.4	12.70	5.11	3.12	2.83
8.07	39.40	20.05	21.61	4.37	5.59	7.49
9.05	46.60	39.05	38.03	7.35	19.39	16.76
13.59	82.91	106.46	134.48	8.00	14.85	21.24
14.15	89.59	119.68	149.43	11.95	23.64	26.74
15.34	107.21	147.52	179.24	14.77	23.34	25.00
17.69	154.13	156.19	189.27	19.99	3.69	4.27
19.09	187.41	177.99	213.38	23.77	15.57	17.22
20.01	213.99	198.74	234.04	28.85	22.52	22.42
22.71	304.20	277.62	301.36	33.43	29.23	24.95
25.42	421.62	453.07	447.94	43.40	64.85	54.18
26.64	479.19	526.35	513.97	47.01	59.84	53.92
29.11	608.82	740.68	725.88	52.48	86.77	85.79
30.45	690.47	800.39	802.02	60.75	44.43	56.65
33.80	949.10	988.09	1027.15	77.29	56.10	67.28
35.57	1121.17	1060.50	1130.08	97.43	41.00	58.28
37.75	1449.05	1121.17	1205.10	150.14	27.78	34.35

**Table 7:** Relation between loads applied and deformation of the rebars 1 and 2.

Load (kN)	Moment (mkN)	Deformation Rebar 2 (µm/m)	Deformation Rebar 1 (µm/m)
5.20	3.90	12.45	9.84
10.41	7.81	14.14	9.94
15.24	11.43	17.10	12.40
20.26	15.20	24.48	17.21
25.11	18.83	28.39	20.20
30.02	22.52	33.77	24.26
35.01	26.26	37.04	26.19
40.32	30.24	45.91	33.03
43.42	32.57	45.27	32.71
54.00	40.50	70.28	50.66
60.54	45.41	252.00	138.00
70.00	52.50	269.20	150.90
80.37	60.28	454.20	306.10
90.00	67.50	612.70	490.10
101.20	75.90	749.70	630.70
110.30	82.73	814.30	700.40
120.80	90.60	900.30	786.80
131.30	98.48	1042.00	925.70
142.10	106.58	1167.00	1055.00
150.00	112.50	1166.00	1056.00
170.50	127.88	1338.00	1233.00
182.10	136.58	1514.00	1401.00
201.20	150.90	1688.00	1559.00
210.00	157.50	1927.00	1783.00
252.20	189.15	2212.00	2066.00
275.20	206.40	2397.00	2255.00

**Table 8:** Deformation of the rebars 1 and 2 for a moment of 127.88 mkN by FAGUS.

Formula/Result	Name	Max.	Min	Value
ε Rebar 1	Rebar 1	0.2058	0.2058	‰
ε Rebar 1 Value in crack	Rebar 1	0.2940	0.2940	‰
ε Rebar 2	Rebar 2	0.2058	0.2058	‰
ε Rebar 2 Value in crack	Rebar 2	0.2940	0.2940	‰

**Table 9:** Theoretical deformation of the rebars 1 and 2 for M_fis_ = 111.25 mkN and f_ct_ = 8.90 MPa with FAGUS.

Load (kN)	Moment (mkN)	Theoretical Deformation (µm/m)	Deformation Rebar 2 (µm/m)	Deformation Rebar 1 (µm/m)
5.20	3.90	6.30	12.45	9.84
10.41	7.81	12.70	14.14	9.94
15.24	11.43	18.50	17.10	12.40
20.26	15.20	24.70	24.48	17.21
25.11	18.83	30.50	28.39	20.20
30.02	22.52	36.50	33.77	24.26
35.01	26.26	42.70	37.04	26.19
40.32	30.24	49.00	45.91	33.03
43.42	32.57	52.90	45.27	32.71
54.00	40.50	65.70	70.28	50.66
60.54	45.41	73.60	252.00	138.00
70.00	52.50	85.10	269.20	150.90
80.37	60.28	97.70	454.20	306.10
90.00	67.50	109.30	612.70	490.10
101.20	75.90	122.90	749.70	630.70
110.30	82.73	133.90	814.30	700.40
120.80	90.60	146.60	900.30	786.80
131.30	98.48	159.40	1042.00	925.70
142.10	106.58	172.30	1167.00	1055.00
150.00	112.50	259.30	1166.00	1056.00
170.50	127.88	294.00	1338.00	1233.00
182.10	136.58	315.90	1514.00	1401.00
201.20	150.90	358.30	1688.00	1559.00
210.00	157.50	380.90	1927.00	1783.00
252.20	189.15	523.10	2212.00	2066.00
275.20	206.40	630.20	2397.00	2255.00

**Table 10:** Deformation of the rebars 1 and 2 for a moment of 127.88 mkN by FAGUS.

Formula/Result	Name	Max.	Min	Value
ε Rebar 1	Rebar 1	1.0294	1.0294	‰
ε Rebar 1 Value in crack	Rebar 1	1.4706	1.4706	‰
ε Rebar 2	Rebar 2	1.0294	1.0294	‰
ε Rebar 2 Value in crack	Rebar 2	1.4706	1.4706	‰

**Table 11:** Deformation of the rebars 1 and 2 for M_fis_ = 40.5 mkN and f_ct_ = 3.24 MPa with FAGUS.

Load (kN)	Moment (mkN)	Deformation without Crack (µm/m)	Cracking Deformation (µm/m)	Interpolation Value (µm/m)	Deformation Rebar 2 (µm/m)	Deformation Rebar 1 (µm/m)
5.20	3.90	6.30		6.30	12.45	9.84
10.41	7.81	12.70		12.70	14.14	9.94
15.24	11.43	18.50		18.50	17.10	12.40
20.26	15.20	24.70		24.70	24.48	17.21
25.11	18.83	30.50		30.50	28.39	20.20
30.02	22.52	36.50		36.50	33.77	24.26
35.01	26.26	42.70		42.70	37.04	26.19
40.32	30.24	49.00		49.00	45.91	33.03
43.42	32.57	52.90		52.90	45.27	32.71
54.00	40.50	64.00	91.50	88.75	70.28	50.66
60.54	45.41	71.80	102.50	99.43	252.00	138.00
70.00	52.50	389.70	556.70	540.00	269.20	150.90
80.37	60.28	460.70	658.10	638.36	454.20	306.10
90.00	67.50	523.90	748.40	725.95	612.70	490.10
101.20	75.90	596.10	851.50	825.96	749.70	630.70
110.30	82.73	653.70	933.90	905.88	814.30	700.40
120.80	90.60	720.10	1028.70	997.84	900.30	786.80
131.30	98.48	786.10	1123.00	1089.31	1042.00	925.70
142.10	106.58	853.40	1219.10	1182.53	1167.00	1055.00
150.00	112.50	902.20	1288.90	1250.23	1166.00	1056.00
170.50	127.88	1029.40	1470.60	1426.48	1338.00	1233.00
182.10	136.58	1101.00	1572.90	1525.71	1514.00	1401.00
201.20	150.90	1218.60	1740.80	1688.58	1688.00	1559.00
210.00	157.50	1272.80	1818.20	1763.66	1927.00	1783.00
252.20	189.15	1532.80	2189.70	2124.01	2212.00	2066.00
275.20	206.40	1870.40	2672.00	2591.84	2397.00	2255.00

**Table 12:** Slope values of theoretical deformation curves and of rebars 1 and 2.

Load (kN)	Interpolation Value (µm/m)	Deformation rebar 2 (µm/m)	Deformation rebar 1 (µm/m)	Slope Interpolation Value	Slope Rebar 2	Slope Rebar 1
5.20	6.30	12.45	9.84	1.23	2.36	1.86
10.41	12.70	14.14	9.94	1.23	0.32	0.02
15.24	18.50	17.10	12.40	1.20	0.61	0.51
20.26	24.70	24.48	17.21	1.24	1.47	0.96
25.11	30.50	28.39	20.20	1.20	0.81	0.62
30.02	36.50	33.77	24.26	1.22	1.10	0.83
35.01	42.70	37.04	26.19	1.24	0.66	0.39
40.32	49.00	45.91	33.03	1.19	1.67	1.29
43.42	52.90	45.27	32.71	1.26	-0.21	-0.10
54.00	88.75	70.28	50.66	3.39	2.36	1.70
60.54	99.43	252.00	138.00	1.63	27.79	13.35
70.00	540.00	269.20	150.90	46.57	1.82	1.36
80.37	638.36	454.20	306.10	9.49	17.84	14.97
90.00	725.95	612.70	490.10	9.10	16.46	19.11
101.20	825.96	749.70	630.70	8.93	12.23	12.55
110.30	905.88	814.30	700.40	8.78	7.10	7.66
120.80	997.84	900.30	786.80	8.76	8.19	8.23
131.30	1089.31	1042.00	925.70	8.71	13.50	13.23
142.10	1182.53	1167.00	1055.00	8.63	11.57	11.97
150.00	1250.23	1166.00	1056.00	8.57	-0.13	0.13
170.50	1426.48	1338.00	1233.00	8.60	8.39	8.63
182.10	1525.71	1514.00	1401.00	8.55	15.17	14.48
201.20	1688.58	1688.00	1559.00	8.53	9.11	8.27
210.00	1763.66	1927.00	1783.00	8.53	27.16	25.45
252.20	2124.01	2212.00	2066.00	8.54	6.75	6.71
275.20	2591.84	2397.00	2255.00	20.34	8.04	8.22
